# Apple U-box-type E3 ubiquitin ligase MdPUB23 reduces cold-stress tolerance by degrading the cold-stress regulatory protein MdICE1

**DOI:** 10.1093/hr/uhac171

**Published:** 2022-08-03

**Authors:** Da-Ru Wang, Xiao-Wei Zhang, Rui-Rui Xu, Gui-Luan Wang, Chun-Xiang You, Jian-Ping An

**Affiliations:** State Key Laboratory of Crop Biology, College of Horticulture Science and Engineering, Shandong Agricultural University, Tai-An, 271018, Shandong, China; State Key Laboratory of Crop Biology, College of Horticulture Science and Engineering, Shandong Agricultural University, Tai-An, 271018, Shandong, China; Key Laboratory of Biochemistry and Molecular Biology in Universities of Shandong, College of Biology and Oceanography, Weifang University, Weifang 261061, Shandong, China; State Key Laboratory of Crop Biology, College of Horticulture Science and Engineering, Shandong Agricultural University, Tai-An, 271018, Shandong, China; State Key Laboratory of Crop Biology, College of Horticulture Science and Engineering, Shandong Agricultural University, Tai-An, 271018, Shandong, China; State Key Laboratory of Crop Biology, College of Horticulture Science and Engineering, Shandong Agricultural University, Tai-An, 271018, Shandong, China

## Abstract

Cold stress limits plant growth, geographical distribution, and crop yield. The MYC-type bHLH transcription factor ICE1 is recognized as the core positive regulator of the cold-stress response. However, how ICE1 protein levels are regulated remains to be further studied. In this study, we observed that a U-box-type E3 ubiquitin ligase, MdPUB23, positively regulated the cold-stress response in apple. The expression of MdPUB23 increased at both the transcriptional and post-translational levels in response to cold stress. Overexpression of *MdPUB23* in transgenic apple enhanced sensitivity to cold stress. Further study showed that MdPUB23 directly interacted with MdICE1, promoting the ubiquitination-mediated degradation of the MdICE1 protein through the 26S-proteasome pathway and reducing the MdICE1-improved cold-stress tolerance in apple. Our results reveal that MdPUB23 regulates the cold-stress response by directly mediating the stability of the positive regulator MdICE1. The PUB23–ICE1 ubiquitination module may play a role in maintaining ICE1 protein homeostasis and preventing overreactions from causing damage to plants. The discovery of the ubiquitination regulatory pathway of ICE1 provides insights for the further exploration of plant cold-stress-response mechanisms.

## Introduction

Plants are constantly subjected to various environmental stimuli during their growth and development, such as extreme temperature, drought, waterlogging, and high salinity [1–3]. To adapt to harsh environmental challenges, plants have evolved elaborate regulatory mechanisms [4–6]. Cold stress is one of the most important abiotic stresses affecting plant growth, geographical distribution, and crop yield. It affects plant metabolism by directly inhibiting the expression of genes related to metabolic enzymes [7–9]. Cold stress rapidly activates the expression of a series of transcription factors, among which C-repeat-binding factors (CBFs) are the most thoroughly studied. They directly induce the expression of downstream cold-responsive (COR) genes, regulating the response to cold stress [7–13]. Three *CBF* genes (*AtCBF1*–*AtCBF3*) were identified in *Arabidopsis*, and five (*MdCBF1*–*MdCBF5*), in apple [14–17]. In *Arabidopsis*, the *CBF* genes are regulated by many upstream transcription factors, including INDUCER OF CBF EXPRESSION1/2 (ICE1/2), CALMODULIN-BINDING TRANSCRIPTION ACTIVATOR1–5 (CAMTA1–5), MYB15, PHYTOCHROME-INTERACTING FACTOR3/4/7 (PIF3/4/7), PSEUDO RESPONSE REGULATORS (PRRs), CIRCADIAN CLOCK-ASSOCIATED1 (CCA1), LATE ELONGATED HYPOCOTYL (LHY), ETHYLENE INSENSITIVE3 (EIN3), CESTA, BRASSINAZOLERESISTANT1 (BZR1), and BRI1-EMS-SUPPRESSOR1 (BES1) [18–32]. ELONGATED HYPOCOTYL5 (MdHY5), MdMYB23, BASIC HELIX–LOOP-HELIX33 (MdbHLH33), and B-box37 (MdBBX37) have been identified as positive regulators of *MdCBF* genes in apple [33–37]. Among them, the ICE1–CBF–COR regulatory module plays a particularly essential role in the cold-stress response [11, 18, 38, 39].

ICE1 interacts with many cold-stress regulatory proteins to jointly regulate the cold-stress response. In *Arabidopsis*, ICE1 directly interacts with MYB15, a negative regulator of cold stress [19]. The jasmonic acid (JA)-signaling repressors JASMONATE ZIM-DOMAIN1 (JAZ1) and JAZ4 interact with ICE1, inhibiting the transcriptional activity of ICE1 [40]. HIGH OSMOTIC EXPRESSION1 (HOS1), MITOGEN-ACTIVATED PROTEIN KINASE3/6 (MPK3/6), and BRASSINOSTEROID-INSENSITIVE2 (BIN2) negatively regulate cold-stress tolerance through interaction with ICE1 and attenuate the protein stability of ICE1 [41–44]. By contrast, SAP and Miz1 (SIZ1) and OPEN STOMATA 1 (OST1) enhance the protein stability of ICE1 in the cold-stress response [28, 45]. Rice OsMAPK3 enhances the stability of OsICE1 by inhibiting OsICE1 degradation via OsHOS1 [46]. In banana fruit, JA-signaling regulators called MaMYC2s may mediate cold-stress tolerance by interaction with MaICE1 [47]. SEVEN IN ABSENTIA1 (MaSINA1) may negatively regulate the cold-stress response by reducing the protein stability of MaICE1 [48]. MdBBX37 and ABSCISIC ACID INSENSITIVE4 (MdABI4), as interaction partners of MdICE1, positively regulate the transcriptional activity of MdICE1 in apple [37, 49].

Post-translational modifications, such as ubiquitination, phosphorylation, methylation, and sumoylation, play key roles in the regulation of protein stability and biological activity [50–54]. Among them, ubiquitination has been well studied. The ubiquitination cascade requires the coordination of three components: E1 ubiquitin-activating enzymes, E2 ubiquitin-conjugating enzymes, and E3 ubiquitin ligases [55, 56]. In particular, E3 ubiquitin ligases play a decisive role in the specificity of target proteins [57, 58]. In *Arabidopsis*, more than 1400 genes encode E3 ubiquitin ligases [57, 59]. According to the characteristic domain of ubiquitin ligases and the mechanism of ubiquitin’s transfer to target proteins, E3 ubiquitin ligases are mainly divided into three categories: homologous to E6AP COOH terminus (HECT)-type E3 ubiquitin ligases, really interesting new gene (RING)-finger-type E3 ubiquitin ligases, and U-box-type E3 ubiquitin ligases [60–62]. Plant U-box-type E3 ubiquitin ligases (PUBs) play a broad role in the regulation of plant growth and development and stress responses [63–65]. In *Arabidopsis*, PUB2/4/12/13/22/23/24/25/26 are involved in plant immune regulation [66–70]. PUB11/22/23/46/48 mediate the drought-stress response [71–74]. PUB10/12/13/18/19/40 play key roles in plant responses to multiple hormonal signals [75–80]. A recent study showed that PUB25 and PUB26 positively regulated the cold-stress response by mediating MYB15 protein degradation [81]. In apples, MdPUB24 and MdPUB29 regulate fruit quality [82, 83]. In addition, MdPUB29 may also be involved in the regulation of the plant immune response to fungal pathogens [84]. However, the roles of PUB proteins in apple remain to be further studied.

In this study, we report that the U-box-type E3 ubiquitin ligase MdPUB23 is a negative regulator of the cold-stress response in apple. MdPUB23 interacts with MdICE1, a key regulator of cold stress, and negatively regulates the cold-stress response by promoting the protein stability of MdICE1. This study reveals a new post-translational regulatory mechanism that maintains the protein stability of ICE1 in the cold-stress response.

## Results

### MdPUB23 interacts with MdICE1

**Figure 1:**
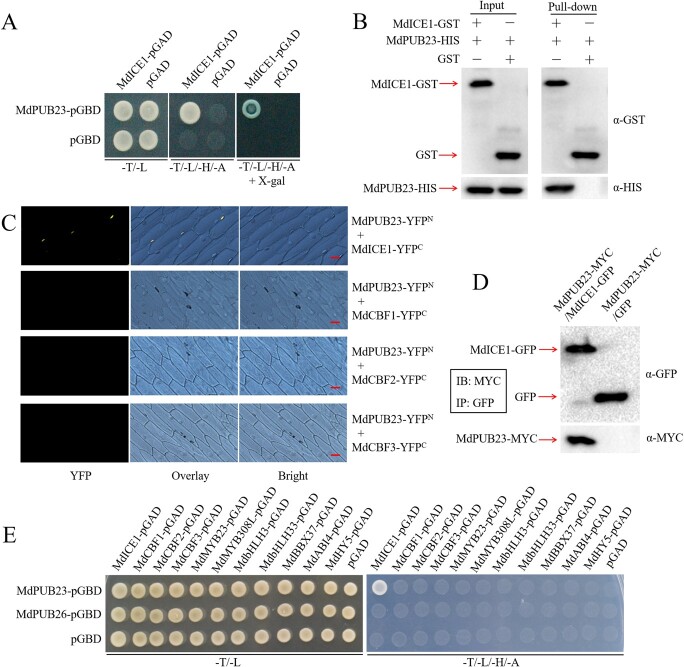
The interaction between MdPUB23 and MdICE1. (**A**) Y2H assays showing the interaction between MdPUB23 and MdICE1 proteins. The full-length *MdICE1* and *MdPUB23* were cloned into a pGAD424 or a pGBT9 vector, respectively. Yeasts grown in SD (-T/-L), SD (-T/-L/-H/-A), and SD (-T/-L/-H/-A + X-gal) media are indicated. (**B**) Pull down assays showing the interaction between MdPUB23 and MdICE1 in vitro. MdPUB23-HIS fusion protein was incubated with a cobalt chelate affinity resin containing the immobilized MdICE1-GST or GST protein. The protein mixtures were purified using a GST purification kit. (**C**) BiFC assays showing the interaction between MdPUB23 and MdICE1 proteins in nuclei of epidermal cells of onions. Bars = 10 μm. (**D**) Co-IP assays showing the interaction between MdPUB23 and MdICE1 proteins *in vivo*. (**E**) Y2H assays showing the interaction between MdPUB23 and a series of cold-stress-responsive proteins. Yeasts grown in SD (-T/-L), SD (-T/-L/-H/-A), and SD (-T/-L/-H/-A + X-gal) media are indicated. All experiments were repeated three times with similar results. A representative picture is shown here.

ICE1 is considered a key positive regulator of the cold-stress response [18, 38]. However, the post-translational regulation of the ICE1 protein has not been fully studied. To study the post-translational regulatory mechanism of MdICE1 in apple, the MdICE1-GFP protein was extracted from *MdICE1*-overexpressing apple calli (MdICE1-GFP) [37] and analysed by mass spectroscopy. The U-box E3 ubiquitin ligase MdPUB23 was isolated as a potential interaction partner of MdICE1. To test the interaction between MdPUB23 and MdICE1, we performed Y2H assays. The results showed that yeast cells transformed with MdPUB23 or MdICE1 alone could not grow on selective medium (−T/−L/−H/−A), and only the yeast cells transformed with MdPUB23 and MdICE1 could grow normally on selective medium (Fig. 1A), suggesting that MdPUB23 and MdICE1 interact with each other in yeast cells. Next, *in vitro* pull-down assays were performed to confirm the interaction. The purified MdPUB23-HIS fusion protein was pulled down using MdICE1-GST (Fig. 1B), indicating that MdPUB23 physically interacted with MdICE1 *in vitro*. We further verified the interaction between MdPUB23 and MdICE1 by BiFC assays. Fluorescence detection results showed that only onion epidermal cells co-expressed MdPUB23 and MdICE1 could produce a strong fluorescence signal (Fig. 1C). These data reveal that MdPUB23 interacts with MdICE1. Moreover, coimmunoprecipitation (Co-IP) assays showed that MdPUB23 interacted with MdICE1 *in vivo* (Fig. 1D). Further Y2H assays showed that MdPUB23 specifically interacted with MdICE1 in yeast cells (Fig. 1E).

**Figure 2:**
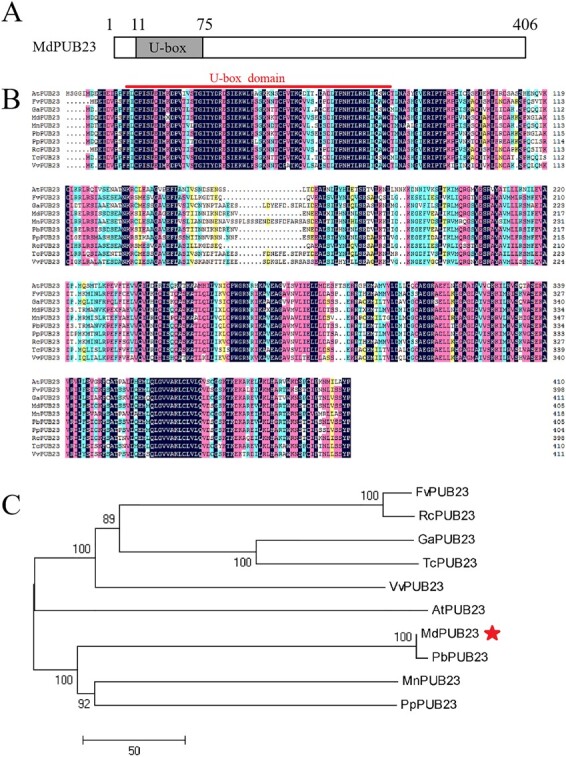
Identification of MdPUB23. (**A**) The structure of the conserved regions of the MdPUB23 protein. The U-box domain (11–75 amino acids) is shown in the black box. (**B**) Multiple sequence alignment of MdPUB23 with PUB23 proteins from different species. PbPUB23, *Pyrus x bretschneideri*, XP_009348934.1; PpPUB23: *Prunus persica*, XP_007204425.1; FvPUB23: *Fragaria vesca subsp. vesca*, XP_004303406.1; RcPUB23: *Rosa chinensis*, XP_024190862.1; MnPUB23: *Morus notabilis*, XP_010106989.1; GaPUB23: *Gossypium arboreum*, XP_017610198.1; TcPUB23: *Theobroma cacao*, XP_007027990.1; VvPUB23: *Vitis vinifera*, XP_002267438.1; MdPUB23: *Malus x domestica*, MDP0000773851; AtPUB23: *Arabidopsis thaliana*, AT2G35930.1. The U-box domain is the red line. (**C**) Phylogenetic tree of above 10 PUB23 proteins (constructed with MEGA 5.0).

**Figure 3:**
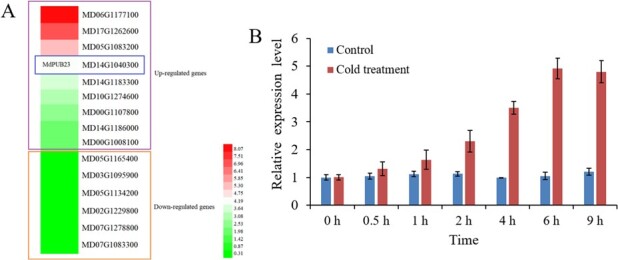
Cold-stress response of MdPUB23. (**A**) Transcriptome analysis showing the expression levels of 15 *PUB* genes after cold-stress treatment in apple. Apple seedlings were exposed to 4°C for 9 h, and seedlings grown at 24°C were used as controls. Total RNAs from plants treated with or without cold stress were extracted and used for transcriptome analyses (An *et al*., 2018a). (**B**) Gene expression analysis of *MdPUB23* under cold-stress treatments. Thirty-day-old apple seedlings grown at 24°C were transferred to 4°C for 9 h and were collected to detect the expression of *MdPUB23* using qRT-PCR. Control, apple seedlings without cold treatment; Cold treatment, apple seedlings with cold treatment. The expression level at 0 h was used as the reference and set to 1. Three biological replicates were carried out with three technical repeats. Error bars denote the standard deviations (SD).

**Figure 4:**
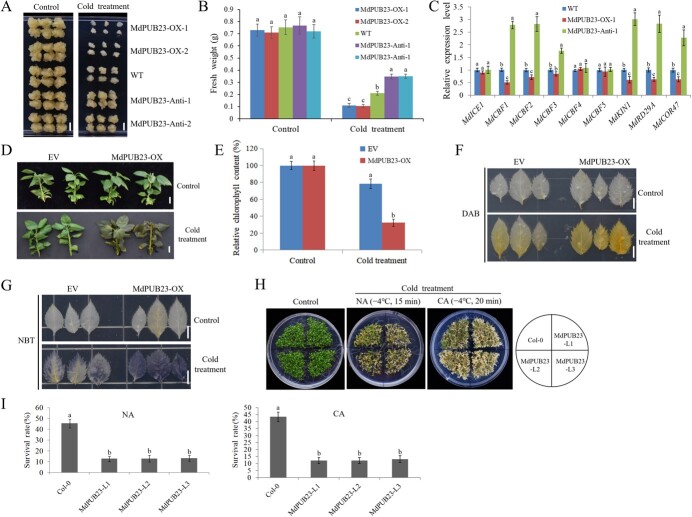
Cold-stress phenotypes of MdPUB23 transgenie apple calli, leaves, and Arabidopsis seedlings. (**A**) Cold-stress phenotypes of MdPUB23 transgenic apple calli. Apple calli were grown on medium at 24°C for 5 d and then treated at 4°C for another 10 d. The experiments were repeated three times with similar results, and each experiment contained 8–C10 calli subgroups per treatment. A representative picture is shown here. WT, wild-type control; MdPUB23-OX-1 and MdPUB23-OX-2, MdPUB23-overexpressing apple calli; MdPUB23-Anti-1 and MdPUB23-Anti-2, apple calli expressing the MdPUB23 antisense construct. Bars = 1 cm. Control, apple calli without cold treatment; Cold treatment, apple calli with cold treatment. (**B**) The fresh weight of apple calli with or without cold stress treatment shown in (**A**). Error bar denotes SD. Data are presented as mean ±C SD of three independent experiments each containing 8–10 calli subgroups per treatment. Different letters above the bars indicate significant differences (p <C 0.05) based on one-way ANOVA with Duncan's test. (**C**) Expression of the MdICE1 and MdCBF genes (MdCBF1, MdCBF2, MdCBF3, MdCBF4, and MdCBF5) and the CBF target genes (MdKIN1, MdRD29A, and MdCOR47) in MdPUB23 transgenic apple calli under cold-stress treatment shown in (**A**). qRT-PCR was performed in three biological replicates and three technical replicates. The value of WT was used as the reference and was set to 1. Error bar denotes SD. Different letters above the bars indicate significant differences (p < 0.05) based on one-way ANOVA with Duncan's test. (**D**) Cold-stress phenotypes of MdPUB23 transient transgenic apple seedlings. Apple seedlings were treated at 4°C for 5 d. The experiments were repeated three times with similar results, and each experiment contained 4–6 seedlings per treatment. A representative picture is shown here. EV, the empty-vector control; MdPUB23-OX, MdPUB23-overexpressing apple seedlings. Bars = 1 cm. Control, apple seedlings without cold treatment; Cold treatment, apple seedlings with cold treatment. (**E**) The relative chlorophyll content of apple seedlings with or without cold-stress treatment shown in (**D**). The value of EV without cold treatment was used as the reference and was set to 100%. Error bar denotes SD. Data are presented as mean ± SD of three independent experiments each containing 4–6 seedlings per treatment. Different letters above the bars indicate significant differences (p < 0.05) based on one-way ANOVA with Duncan's test. (**F**) DAB and (**G**) NBT staining of MdPUB23 transient transgenic apple leaves. Apple leaves were treated at 4°C for 10 h, and then, leaves were dyed with DAB and NBT. EV, the empty-vector control; MdPUB23-OX, MdPUB23-overexpressing apple leaves. Control, apple leaves without cold treatment; Cold treatment, apple leaves with cold treatment. The experiments were repeated three times with similar results, and each experiment contained 6–C9 apple leaves per treatment. Bars = 1 cm. A representative picture is shown here. (**H**) Cold-stress phenotypes of MdPUB23 transgenic Arabidopsis seedlings. Arabidopsis seedlings were grown on MS plates at 22°C for 8 d and then treated at −4°C for 15 min for non-acclimated plants (NA) and at −4°C for 20 min for acclimated plants (CA: 3 d at 4°C). Plants without cold-stress treatment were used as control. The experiments were repeated three times with similar results, and each experiment contained 3–5 plates of Arabidopsis seedlings per treatment. A representative picture is shown here. Col-0, wild-type control; MdPUB23-L1, L2, and L3, MdPUB23-overexpressing Arabidopsis seedlings. (**I**) Survival rates of MdPUB23 transgenic Arabidopsis seedlings under non-acclimated and acclimated conditions shown in (**H**). Error bar denotes SD. Data are presented as mean ± SD of three independent experiments each containing 3–5 plates of Arabidopsis seedlings per treatment. Different letters above the bars indicate significant differences (p <C 0.05) based on one-way ANOVA with Duncan's test.

**Figure 5:**
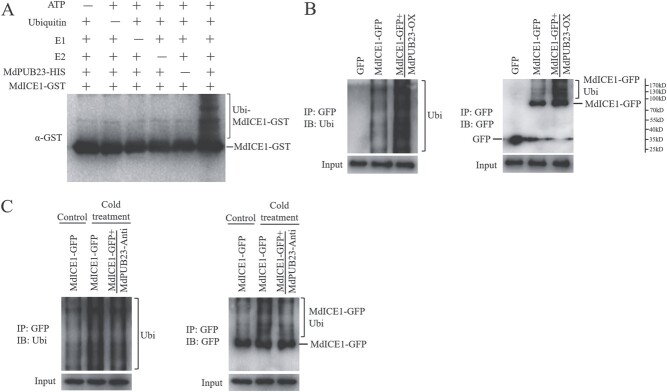
Effect of MdPUB23 on level of ubiquitination of MdICE1. (**A**) Ubiquitination assays *in vitro*. MdPUB23-HIS was tested for E3 ubiquitin ligase activity in the presence and absence of ATP, ubiquitin, E1, E2, MdPUB23-HIS, and MdICE1-GST. The protein gel blot was analyzed using a GST antibody. (**B**) Ubiquitination analysis *in vivo*. MdICE1-GFP was immunoprecipitated using a GFP antibody from the three transgenic apple calli (GFP, MdICE1-GFP, and MdICE1-GFP/MdPUB23-OX). Immunoblotting using a ubiquitin (Ubi) antibody is shown on the left, and that using a GFP antibody is shown on the right. HIS fusion protein was used as the control. (**C**) Effect of cold stress on level of ubiquitination of MdICE1. MdICE1-GFP was immunoprecipitated using a GFP antibody from the two transgenic apple calli (MdICE1-GFP and MdICE1-GFP/MdPUB23-Anti) with or without cold-stress treatment for 1 h. Immunoblotting using a ubiquitin (Ubi) antibody is shown on the left, and that using a GFP antibody is shown on the right. Control, without cold-stress treatment; Cold treatment, with cold-stress treatment.

**Figure 6:**
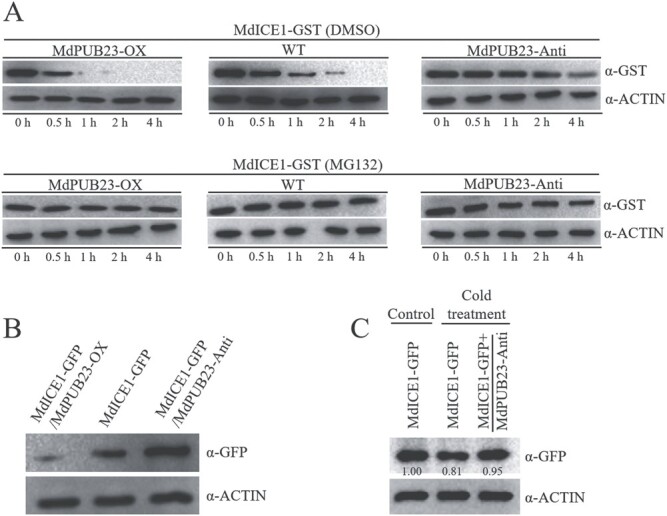
Effect of MdPUB23 on the stability of MdICE1 protein. (**A**) Protein-degradation assays *in vitro*. Total proteins extracted from wild-type (WT) and *MdPUB23* transgenic apple calli (MdPUB23-OX: MdPUB23 overexpression; MdPUB23-Anti: *MdPUB23* antisense suppression) with or without 100 μM MG132 treatments were incubated with the purified MdICE1-GST fusion protein. The samples were collected at the indicated times (0, 0.5, 1, 2, and 4 h). ACTIN was used as an internal reference. (**B**) MdICE1-GFP protein abundance in transgenic apple calli (MdICE1-GFP, MdICE1-GFP/MdPUB23-OX, and MdICE1-GFP/MdPUB23-Anti) was assessed by immunoblotting using a GFP antibody. (**C**) Effect of cold stress on the stability of MdICE1 protein. MdICE1-GFP and MdICE1-GFP/MdPUB23-Anti transgenic apple calli were treated with or without cold stress for 1 h. MdICE1-GFP protein abundance was assessed by immunoblotting using a GFP antibody. Control, without cold-stress treatment; Cold treatment, with cold-stress treatment.

**Figure 7:**
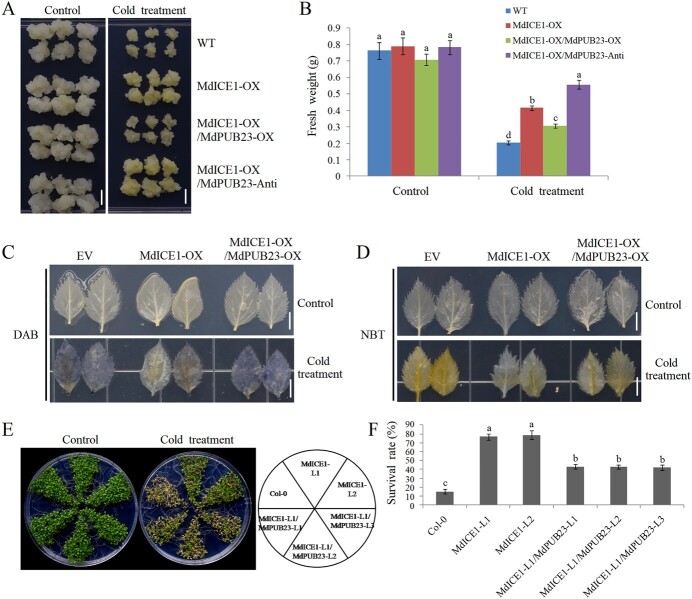
Effects of MdPUB23 on MdICE1’s regulation of cold tolerance. (**A**) Cold-stress phenotypes of MdICE1-OX/MdPUB23-OX transgenic apple calli. WT, wild-type control; MdICE1-OX, *MdICE1*-overexpressing calli; MdICE1-OX/MdPUB23-OX, overexpression of *MdPUB23* in the background of *MdICE1*-overexpressing calli; MdICE1-OX/MdPUB23-Anti, suppression of *MdPUB23* in the background of *MdICE1*-overexpressing calli. Control, apple calli without cold treatment; Cold treatment, apple calli with cold treatment. Apple calli were grown on medium at 24°C for 5 d and then treated at 4°C for another 10 d. The experiments were repeated three times, and each experiment contained 8–10 calli subgroups per treatment. Bars = 1 cm. A representative picture is shown here. (**B**) The fresh weight of apple calli with or without cold-stress treatment shown in (**A**). Error bar denotes SD. Data are presented as mean ± SD of three independent experiments each containing 8–10 calli subgroups per treatment. Different letters above the bars indicate significant differences (*p* < 0.05) based on one-way ANOVA with Duncan's test. (**C**) DAB and (**D**) NBT staining of *MdICE1* and *MdPUB23* transient co-expressing apple leaves. Apple leaves were treated at 4°C for 1 d, and then, leaves were dyed with DAB and NBT. EV, the empty-vector control; MdICE1-OX, MdICE1-overexpressing apple leaves; MdICE1–OX/MdPUB23–OX, MdICE1 and MdPUB23 co-expressing apple leaves. Control, apple leaves without cold treatment; Cold treatment, apple leaves with cold treatment. The experiments were repeated three times with similar results, and each experiment contained 6–9 apple leaves per treatment. Bars = 1 cm. A representative picture is shown here. (**E**) Cold-stress phenotypes of *MdICE1* or *MdICE1–MdPUB23* transgenic *Arabidopsis* seedlings. Col-0, wild-type control; MdICE1-L1 and L2, *MdICE1*-overexpressing *Arabidopsis* seedlings; MdICE1-L1/MdPUB23-L1, MdICE1-L1/MdPUB23-L2, and MdICE1-L1/MdPUB23-L3, *MdICE1* and *MdPUB23* co-transformed *Arabidopsis*. Arabidopsis seedlings were grown on MS plates at 22°C for 8 d and then treated at −4°C for 1 h. The experiments were repeated three times, and each experiment contained 3–5 plates of Arabidopsis seedlings per treatment. A representative picture is shown here. (**F**) Survival rates of *MdICE1* or *MdICE1–MdPUB23* transgenic* Arabidopsis* seedlings shown in (**E**). Error bars denote standard deviations. Different letters above the bars indicate significant differences (*p* < 0.05) as obtained by one-way ANOVA tests.

**Figure 8:**
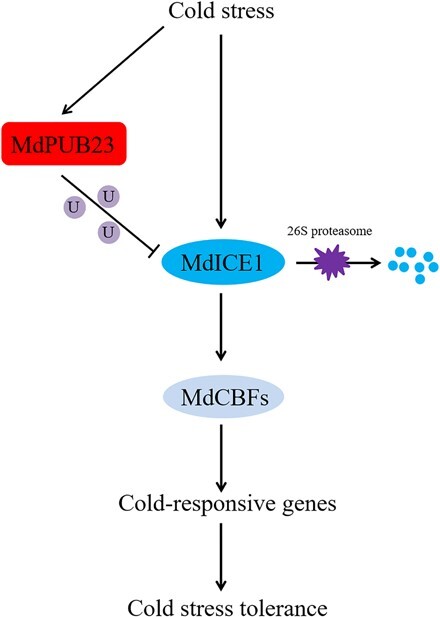
A hypothetical model of the role of MdPUB23 in cold-stress response. MdPUB23 degraded the MdICE1 protein through the ubiquitination pathway and reduced cold resistance. Under cold-stress conditions, MdPUB23 was inhibited and the MdICE1 protein was released from the MdPUB23–MdICE1 ubiquitin module. MdICE1 enhanced cold resistance through the ICE1–CBF–COR pathway.

### Identification of MdPUB23 encoding a U-box E3 ligase

Identification and amino acid sequence analysis revealed that the cDNA sequence of *MdPUB23* comprised 1221 bp and encoded 406 amino acids with a predicted U-box motif in the N-terminal region (Fig. 2A). MdPUB23 showed high sequence similarity with PUB23 proteins in nine other species, especially the conserved U-box motif (Fig. 2B). Among them, MdPUB23 shared the lowest sequence identity with AtPUB23 (58.07%) and the highest sequence identity with PbPUB23 (97.04%). The phylogenetic tree analysis showed that MdPUB23 and PbPUB23 had the closest relationship (Fig. 2C), suggesting that there may be functional similarities between the two.

### MdPUB23 reduces cold-stress tolerance

Because MdPUB23 acts as an interaction partner of MdICE1, we hypothesized that MdPUB23 might be involved in the cold-stress response. To confirm this hypothesis, we first detected the expression of MdPUB23 in response to cold stress. Previous transcriptome data showed that cold stress induced *MdPUB23* expression (Fig. 3A) [34]. qRT-PCR results showed that the expression of *MdPUB23* increased gradually after cold-stress treatment (4°C), and the expression was the highest after 6 h of cold-stress treatment (Fig. 3B). These results demonstrate that cold stress promotes the expression of MdPUB23.

To identify the biological function of MdPUB23 in the cold-stress response, we generated stable *MdPUB23* transgenic apple calli (MdPUB23-OX-1 and MdPUB23-OX-2, *MdPUB23*-overexpressing apple calli; MdPUB23-Anti-1 and MdPUB23-Anti-2, apple calli expressing the *MdPUB23* antisense construct; Supplementary Fig. S1A). Although there was no significant difference in the growth status of wild-type (WT) and *MdPUB23* transgenic apple calli at room temperature (24°C), after cold-stress treatment, the overexpression of *MdPUB23* inhibited the growth of calli compared with the WT control, while the suppression of *MdPUB23* expression contributed to the growth of calli (Fig. 4A–B). Gene expression analysis showed that the overexpression of *MdPUB23* inhibited and the suppression of *MdPUB23* increased the expression of *MdCBF1* and *MdCBF2* and its target genes (Fig. 4C). To evaluate its function in intact plant tissues, transient transgenic apple seedlings and leaves overexpressing *MdPUB23* were generated and subjected to cold-stress treatment (Supplementary Fig. S1B, C).

After cold-stress treatment, the chlorophyll loss of transgenic apple seedlings was determined, and the transgenic apple leaves were stained with reactive oxygen species (ROS) dye to determine the degree of damage to the seedlings or leaves caused by the cold stress. The results showed that, compared with the empty-vector control (EV), the chlorophyll loss and ROS content in the *MdPUB23* transgenic leaves were higher after cold-stress treatment (Fig. 4D–G), indicating that MdPUB23 negatively regulates cold-stress tolerance. Furthermore, we obtained *MdPUB23* transgenic *Arabidopsis* seedlings and used them for cold-stress assays (Supplementary Fig. S1D). The survival rate of *MdPUB23*-overexpressing *Arabidopsis* was lower than that of the Col-0 after cold-stress treatment (Fig. 4H, I). These results indicate that MdPUB23 negatively regulates cold-stress tolerance.

### MdPUB23 mediates the ubiquitination of MdICE1 *in vitro* and *in vivo*

Given that MdPUB23 encodes a E3 ubiquitin ligase and MdICE1 acts as an interaction partner of MdPUB23, we questioned whether MdPUB23 could mediate the ubiquitination of the MdICE1 protein. *In vitro* ubiquitination assays showed that only when ATP, ubiquitin, E1, E2, and MdPUB23-HIS were present at the same time would ubiquitin-modification bands appear for MdICE1-GST (Fig. 5A), suggesting that MdICE1 is a direct target of MdPUB23 ubiquitination. In addition, we detected the ubiquitination of MdICE1 by MdPUB23 *in vivo*. Apple calli of single transgenic *MdICE1* and co-transgenic *MdICE1* and *MdPUB23* were prepared (Supplementary Fig. S1A and E). The MdICE1-GFP protein was immunocoprecipitated with GFP antibody and detected with GFP and ubiquitin antibodies. The results showed that the overexpression of *MdPUB23* significantly increased the degree of polyubiquitination of MdICE1 (Fig. 5B), demonstrating that MdPUB23 mediates the ubiquitination of MdICE1 *in vivo*. Moreover, we found that cold stress enhanced MdICE1 ubiquitination and this process was dependent on MdPUB23 (Fig. 5C).

### MdPUB23 promotes MdICE1 degradation

To assess whether MdPUB23 affected the protein stability of MdICE1, *in vitro* protein-degradation assays were performed. The MdICE1-GST fusion protein was incubated with total proteins extracted from WT and *MdPUB23* transgenic apple calli. Western blotting analysis showed that the degradation rate for MdICE1-GST was higher in *MdPUB23*-overexpressing calli than in the WT control, while the suppression of *MdPUB23* expression reduced the degradation rate for the MdICE1 (Fig. 6A). When the proteasome inhibitor MG132 was applied, degradation of MdICE1 was completely abolished (Fig. 6A), indicating that MdPUB23 promotes MdICE1 degradation via the 26S-proteasome pathway. Next, we detected the protein abundance of MdICE1 in the *MdICE1* single and *MdICE1*–*MdPUB23* co-transformed apple calli using a GFP antibody. The results showed that the overexpression of *MdPUB23* decreased, while the suppression of *MdPUB23* increased the abundance of the MdICE1 protein (Fig. 6B). These results suggest that MdPUB23 targets MdICE1 for ubiquitinated degradation. Moreover, we found that cold stress promoted MdICE1 degradation and this process was dependent on MdPUB23 (Fig. 6C and Supplementary Fig. S2).

### MdPUB23 negatively regulates MdICE1-induced increase in cold-stress tolerance

Because MdPUB23 mediates the ubiquitin degradation of MdICE1 and negatively regulates cold-stress tolerance, we further investigated whether MdPUB23 played a role in MdICE1-mediated cold-stress resistance. Apple calli co-transformed for *MdICE1* and *MdPUB23* were prepared and used for cold-stress assays (Supplementary Fig. 1E). After cold-stress treatment, the overexpression of *MdPUB23* inhibited the growth of calli compared with the WT control, while the suppression of *MdPUB23* expression contributed to the growth of calli (Fig. 4A–B). After cold-stress treatment, the overexpression of *MdICE1* increased calli growth compared with the WT control, which was consistent with previous research results (Fig. 7A, B) [37]. The overexpression of *MdPUB23* on the basis of MdICE1-OX inhibited MdICE1-mediated cold resistance, while the suppression of *MdPUB23* expression further improved MdICE1-induced cold resistance (Fig. 7A–B), suggesting that MdPUB23 negatively regulates MdICE1-increased cold-stress tolerance. In parallel, we generated apple leaves and *Arabidopsis* seedlings co-transformed with *MdICE1* and *MdPUB23* (Supplementary Fig. 1F and G). As expected, cold-stress assays showed that the overexpression of *MdPUB23* reduced MdICE1-induced cold-stress tolerance in apple leaves and *Arabidopsis* seedlings (Fig. 7C–F). Taking these data together, we conclude that MdPUB23 negatively regulates MdICE1-induced cold-stress tolerance by targeting MdICE1 for ubiquitinated degradation.

## Discussion

As one of the most productive and consumed fruits in the world, apples are popular for their rich nutritional value. In the process of apple cultivation and management, extreme-low-temperature disasters result in inestimable losses in fruit-tree production. Preventing and reducing the harm to fruit trees caused by extreme low temperatures is a prerequisite for stable and high yields for fruit trees. Therefore, it is of great practical significance to study the mechanism of the response to low-temperature stress in apple. In the present study, an apple U-box-type E3 ubiquitin ligase, MdPUB23, induced by cold stress at the transcriptional level (Fig. 3), acted as a negative regulator of the cold-stress response (Fig. 4).

ICE1 is recognized as a key regulator of the cold-stress response that enhances the cold-stress resistance of plants by directly mediating the expression of *CBF*s [18,38, 39]. The ICE1–CBF module plays an important role in the regulation of plant growth and development and the cold-stress response [11, 38, 39]. As a core regulator of the cold-stress response, the protein abundance of ICE1 remains dynamically stable in plants. In *Arabidopsis*, the protein kinases MPK3/6 and BIN2 negatively regulate the stability of the ICE1 protein, whereas OST1 increases the stability of ICE1 [28, 42–44]. In addition, the SUMO E3 ligase SIZ1 also enhances the protein stability of ICE in the cold-stress response [45]. In addition to phosphorylation and sumoylation, ubiquitination plays an essential role in the regulation of protein stability and activity of ICE1. The E3 ubiquitin ligase HOS1 targets ICE1 for degradation, thus negatively regulating the cold-stress response in *Arabidopsis* and rice [41, 46]. The banana fruit SINA E3 ubiquitin ligase MaSINA1 interacts with MaICE1 and attenuates its protein stability, reducing cold-stress tolerance [48]. Besides HOS1 and SINA1, no other E3 ubiquitin ligases have been found to regulate the protein stability of ICE1. Here, we used MdICE1 as the bait protein to obtain an E3 ubiquitin ligase, MdPUB23, which may be an interaction partner of MdICE1. The direct interaction between MdPUB23 and MdICE1 was verified by Y2H, pull-down, and BiFC assays (Fig. 1).

PUBs encode a class of E3 ubiquitin ligase proteins characterized by a specific U-box domain [65, 85]. As a typical U-box protein, MdPUB23 contains a U-box motif in the N-terminal region (Fig. 2A). Sequence alignment and phylogenetic tree analysis showed that MdPUB23 shared the highest sequence identity and the closest relationship with PbPUB23 (Fig. 2B–C). PUB E3 ubiquitin ligases are involved in the regulation of multiple stress responses, including the cold-stress response [63–65]. OsPUB2 and OsPUB3 positively regulate the low-temperature-stress response in rice [86]. In *Vitis pseudoreticulata*, VvPUB24 enhances cold-stress tolerance by alleviating the ubiquitin degradation of VvICE1 by VvHOS1 [87]. A recent study showed that *Arabidopsis* PUB25 and PUB26 enhanced the cold-stress response by promoting the degradation of MYB15, a negative regulator of cold stress [81]. However, the mechanism of the PUB-mediated cold-stress response remains unclear, especially in apple. Different from the results for *V. pseudoreticulata* showing that VvPUB24 interacts with VvICE1 but cannot directly regulate the stability of VvICE1 [87], we found that MdPUB23 directly interacted with MdICE1 to promote the ubiquitination degradation of MdICE1 in apple, thus negatively regulating MdICE1-induced cold-stress tolerance (Figs 5, 6 and 7), which suggests that the functions and regulatory mechanisms of proteins in the same family may be also different in different species. Previous studies on the functions of PUB23 have focused on plant immunity and drought stress [66, 71, 73]. The study of PUB23’s involvement in the cold-stress response in this work will further enrich the knowledge of its biological functions.

A hypothetical model is proposed to demonstrate the role of MdPUB23 in the cold-stress response (Fig. 8). Under cold-stress conditions, MdICE1 enhances cold-stress tolerance through the ICE1–CBF–COR transcription cascade pathway. In addition, cold stress can also promote the expression of MdPUB23, which targets the MdICE1 protein for degradation through the 26S-proteasome pathway, thus maintaining ICE1-protein homeostasis and preventing overreactions from causing damage to plants. This study reveals the ubiquitination regulation of the ICE1 protein, providing new insights for further enriching knowledge on and studying the regulatory pathways of plant cold-stress responses.

## Materials and methods

### Plant materials

The plant materials used in this study included apple calli (*Malus domestica*, Orin), apple tissue culture seedlings (*M. domestica*, GL-3), and *Arabidopsis* seedlings (*Arabidopsis thaliana*, Col-0). The detached apple leaves were collected from apple tissue culture seedlings. The growth conditions of plant materials can be queried in previous studies in our laboratory [33].

### Vector construction and genetic transformation

*MdICE1* and *MdPUB23* genes were cloned by using PCR technology from the apple tissue culture seedlings GL-3. To construct the *MdICE1* and *MdPUB23* overexpression recombinant plasmids, full-length *MdICE1* and *MdPUB23* were cloned into pCXSN-GFP and pRI101 vectors, respectively. The fragment of *MdPUB23* was cloned into the pCXSN vector to construct the *MdPUB23* suppression expression recombinant plasmid. Transgenic apple calli and leaves were obtained as previously described in our laboratory [37]. The primers used in this study are listed in Supplementary Table S1.

### Screening the interacting proteins

The MdICE1-GFP protein was extracted from *MdICE1*-overexpressing apple calli and analysed by mass spectroscopy to screen the MdICE1-interacting proteins [88].

### Gene expression analysis

TRIzol RNA extraction solution (Thermo Fisher Scientific, Waltham, MA, USA) and a PrimeScript™ RT kit (Takara, Dalian, China) were used for RNA extraction and reverse transcription, respectively, as previously described in our laboratory [88]. qRT-PCR analysis was performed to determine the expression levels of cold-stress-responsive genes.

### Y2H, pull-down, BiFC, and co-IP assays

The interaction between MdPUB23 and MdICE1 was studied by Y2H, pull-down, BiFC, and Co-IP assays. Detailed experimental methods can be found in the Supplementary Experimental Procedures 1–4.

### Cold-stress assays

Cold-stress assays of apple calli, detached apple leaves, and *Arabidopsis* seedlings were performed as previously described in our laboratory [37]. After cold-stress treatment, the fresh weight of the calli, reactive oxygen species (ROS) staining of the leaves, and survival rate of the *Arabidopsis* seedlings were determined.

### ROS staining

The ROS of apple leaves after cold-stress treatment were dyed with diaminobenzidine (DAB) and nitroblue tetrazolium (NBT) as previously described in our laboratory [37].

### *In vitro* ubiquitination and protein-degradation assays

*In vitro* ubiquitination and protein-degradation assays were performed as previously described in our laboratory [37].

### Accession numbers

MdPUB23, MD14G1040300 (MDP0000773851); MdPUB26, MDP0000448457; MdICE1, MDP0000662999; MdCBF1, HM992942; MdCBF2, MDP0000198054; MdCBF3, Genomic position: MDC023575.38:2048.0.2752; MdCBF4, MDP0000154764; MdCBF5, Genomic position: MDC001207.483:32385.0.33047; MdMYB23, MDP0000230141; MdMYB308L, MDP0000950559; MdbHLH3, MDP0000225680; MdbHLH33, MDP0000309179; MdBBX37, MDP0000157816; MdABI4, MD01G1155400; MdHY5, MDP0000586302; MdKIN1, MDP0000165526; MdRD29A, MDP0000598443; MdCOR47, MDP0000529003; MdACTIN, EB136338.

## Acknowledgements

This work was financially supported by grants from the China postdoctoral Science Foundation (2022 M710086), the Natural Science Foundation of China (32172538), and the Open Project Programme of the State Key Laboratory of Crop Biology (2021KF06).

## Author contributions

J.-P.A. conceived and designed the experiments. D.-R.W. and J.-P.A. performed the research. X.-W.Z., R.-R.X., G.-L.W., and C.-X.Y. analysed the data. J.-P.A. wrote the paper.

## Data availability

All the data generated or analysed during this study are included in this published article.

## Conflict of interest

The authors declare no competing interests.

## Supplementary data

Supplementary data is available at *Horticulture Research * online.

## Supplementary Material

Web_Material_uhac171
