# Supplementary material for: Apple U-box-type E3 ubiquitin ligase MdPUB23 reduces cold-stress tolerance by degrading the cold-stress regulatory protein MdICE1
Source: Hortic Res. 2022 Aug 3;9:uhac171. doi: 10.1093/hr/uhac171 (PMC9557189; doi:10.1093/hr/uhac171)
Supplement: Web_Material_uhac171 [file web_material_uhac171.pdf]

Supplementary Figures

Figure S1. Identification of the transgenic plant materials.

(A) qRT-PCR analysis the expression of *MdPUB23* in transgenic apple calli. WT: wild type apple calli; MdPUB23-OX-1 and MdPUB23-OX-2: *MdPUB23*-overexpressing apple calli; MdPUB23-Anti-1 and MdPUB23-Anti-2: *MdPUB23*-antisense suppressing apple calli; MdICE1-OX/MdPUB23-OX: overexpression of *MdPUB23* in the background of *MdICE1*-overexpressing apple calli; MdICE1-OX/MdPUB23-Anti: antisense suppression of *MdPUB23* in the background of *MdICE1*-overexpressing apple calli. The value for WT was used as the reference and was set to 1. (B) qRT-PCR analysis the expression of *MdPUB23* in transgenic apple leaves. EV: empty vector control; MdPUB23-OX: *MdPUB23*-overexpressing apple leaves; MdICE1-OX/MdPUB23-OX: overexpression of *MdPUB23* in the background of *MdICE1*-overexpressing apple leaves. The value for EV was used as the reference and was set to 1. (C) qRT-PCR analysis the expression of *MdPUB23* in transgenic apple seedlings. EV: empty vector control; MdPUB23-OX: *MdPUB23*-overexpressing apple seedlings. The value for EV was used as the reference and was set to 1. (D) qRT-PCR analysis the expression of *MdPUB23* in transgenic *Arabidopsis* seedlings. Col-0: wild-type *Arabidopsis*; MdPUB23-L1, L2, and L3: *MdPUB23* ectopic transgenic *Arabidopsis*. The value for Col-0 was used as the reference and was set to 1. (E) qRT-PCR analysis the expression of *MdICE1* in transgenic apple calli. WT: wild type apple calli; MdICE1-OX: *MdICE1*-overexpressing apple calli; MdICE1-OX/MdPUB23-OX: overexpression of *MdPUB23* in the background of *MdICE1*-overexpressing apple calli; MdICE1-OX/MdPUB23-Anti: antisense suppression of *MdPUB23* in the background of *MdICE1*-overexpressing apple calli. The value for WT was used as the reference and was set to 1. (F) qRT-PCR analysis the expression of *MdICE1* in transgenic apple leaves. EV: empty vector control; MdICE1-OX: *MdICE1*-overexpressing apple leaves; MdICE1-OX/MdPUB23-OX: overexpression of *MdPUB23* in the background of *MdICE1*-overexpressing apple leaves. The value for EV was used as the reference and was set to 1. (G) qRT-PCR analysis the expression of *MdICE1* in transgenic *Arabidopsis* seedlings. Col-0: wild-type *Arabidopsis*; MdICE1-L1 and L2: *MdICE1* ectopic transgenic *Arabidopsis*. The value for Col-0 was used as the reference and was set to 1. The error bar is the standard deviation.

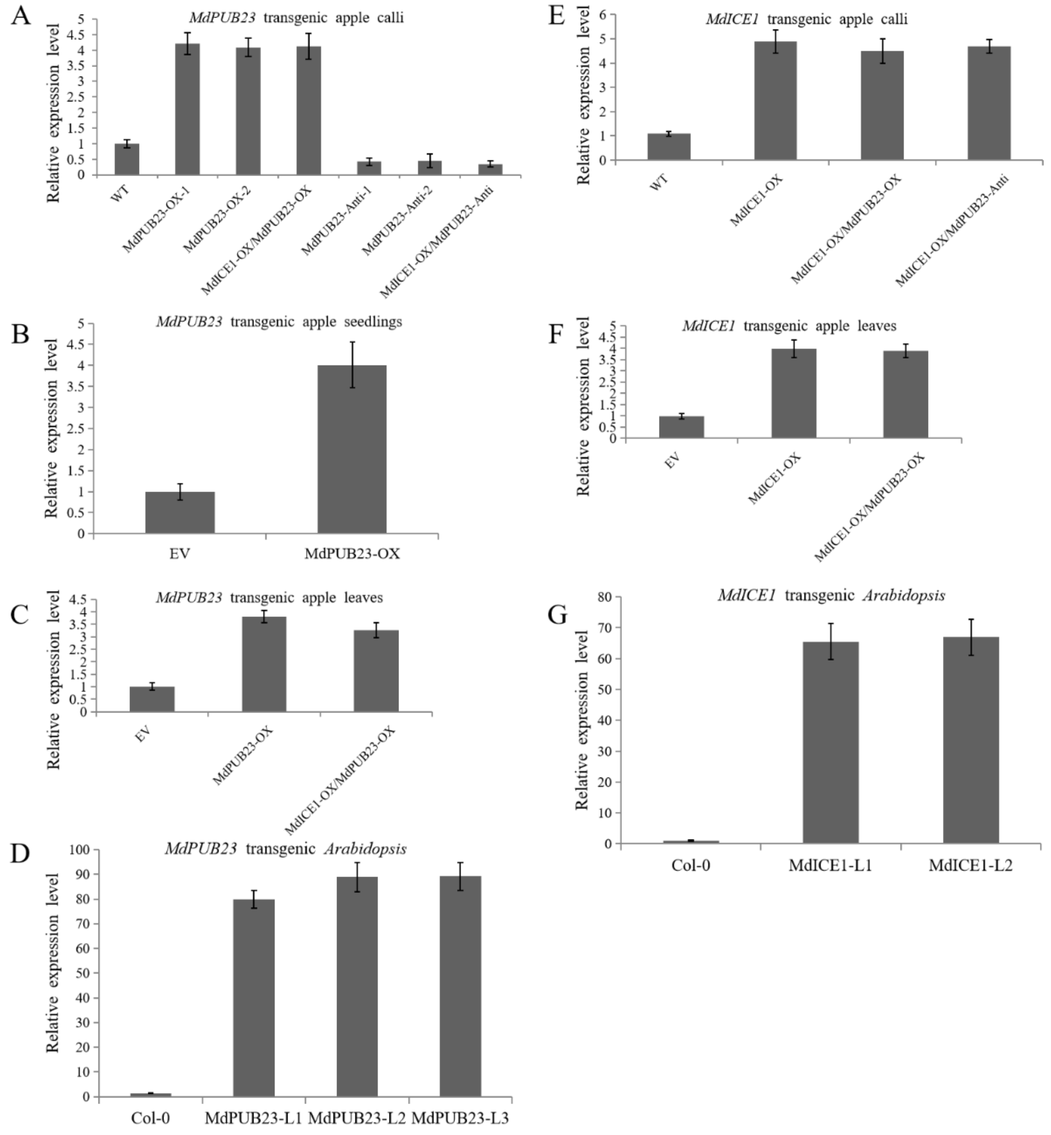

Figure S2. Effects of cold stress on the stability of MdICE1 protein.
Total proteins extracted from wild-type (WT) and *MdPUB23* antisense suppression apple calli with or without cold-stress treatment were incubated with the purified MdICE1-GST fusion protein. The samples were collected at the indicated times (0, 0.5, 1, 2, and 4 h). ACTIN was used as an internal reference.

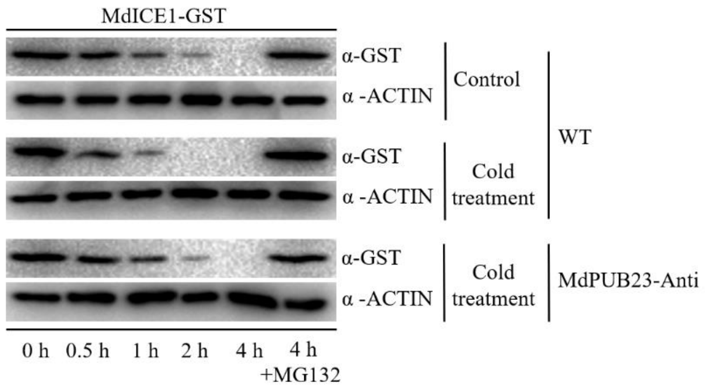

Supplemental Tables

Table S1. Primers used for gene expression analysis and vector construction

Primer name	sequence (5' to 3')
MdPUB23-F	ATGGACCAAGAAATCGACGT
MdPUB23-R	AGATGGATAAGAAGAAACCA
asMdPUB23-F	AGCAGGGGCGGTTATGGTCTTG
asMdPUB23-R	TGAAGAACTAAGCACAGCTTCG
MdICE1-F	ATGCTGCCAAGGCTGAACGG
MdICE1-R	CATCATGCCATGGAACCCGA
MdCBF1-F	ATGGATGGTTGCTCTAATTA
MdCBF1-R	AATGGAAAAACTCCATAAGG
MdCBF2-F	ATGAATACGATCTTCAGTAC
MdCBF2-R	ATTTGAGAAGCTCCACAGAC
MdCBF3-F	ATGAATACGATCTTCAGTCA
MdCBF3-R	ATTTGAGAAGCTCCACAGAC
MdMYB23-F	ATGGGGAGAGCTCCTTGCTG
MdMYB23-R	AAATTCTGGTAATTCTGGCG
MdMYB308L-F	ATGGGAAGATCTCCTTGCTG
MdMYB308L-R	TTTCATCTCCAAGCTTCTGT
MdbHLH3-F	ATGGCTGCACCGCCGCCAAGCA
MdbHLH3-R	AGAGTCAGATTGGGGTATAATTT
MdbHLH33-F	ATGGCTCAGAATCATGAGAG
MdbHLH33-R	ATTTTGAACACGTGAAGATG
MdBBX37-F	ATGAAGAAAGTTTACAGGGC
MdBBX37-R	ACACTCCGCCCAGCCTTCCTCC
MdABI4-F	ATGGAAGAAGACCAACACCGC
MdABI4-R	TGAATCCAATCCCTTAAAATC
MdHY5-F	ATGCAAGAGCAGGCGACGAG
MdHY5-R	ATCCGCATTTGCACCACCAT
MdPUB23(qRT)-F	AGCAGGGGCGGTTATGGTCTTG
MdPUB23(qRT)-R	TGAAGAACTAAGCACAGCTTCG
MdICE1(qRT)-F	ACCGGTGGAGGAGGGGGTCA
MdICE1(qRT)-R	CCTTGCAGCTTGGCCATTTG
MdCBF1(qRT)-F	GAGGAGTCGGATGAGGTTTGT
MdCBF1(qRT)-R	CCACGTCATCATCATCACTGTA
MdCBF2(qRT)-F	GGAGGCTGCAGAGACGTTC
MdCBF2(qRT)-R	CAGCAACCTTGGCATGTCG
MdCBF3(qRT)-F	GAGGTCATTTTGGCGTCCA
MdCBF3(qRT)-R	TGGTTCTCTCATTTCGCACAC
MdCBF4(qRT)-F	TGCGAAATGAGAGAACCAAACA
MdCBF4(qRT)-R	GCGGAGTCTGCAAAATTGAG
MdCBF5(qRT)-F	AAGAGTCGGATGAGGCTTGTG
MdCBF5(qRT)-R	CATTCCTTCCGCCATATTTG
MdKIN1(qRT)-F	ATTTCGGATTATGGATACCAAGTTG

MdKIN1(qRT)-R	TAACGAACACGTACAGATTGCCTTT
MdRD29A(qRT)-F	ACGAGAAGGTTGCCGGGGCAGGGAG
MdRD29A(qRT)-R	GGCACCGCTCCTAGGTTGCTCTGCC
MdCOR47(qRT)-F	AGAAGAAGCAGAGGAAAGAAAAGA
MdCOR47(qRT)-R	CTCCTCAGTCTTCTTGTGGCCAGGA
MdACTIN(qRT)-F	ACACGGGGAGGTAGTGACAA
MdACTIN(qRT)-R	CCTCCAATGGATCCTCGTTA

Supplementary experimental procedures

Experimental procedure S1

Yeast two-hybrid (Y2H) assays

To construct MdPUB23-pGBD and MdPUB26-pGBD, full-length *MdPUB23* and *MdPUB26* were amplified and cloned into the pGBT9 vector. Full-length *MdICE1*, *MdCBF1*, *MdCBF2*, *MdCBF3*, *MdMYB23*, *MdMYB308L*, *MdbHLH3*, *MdbHLH33*, *MdBBX37*, *MdABI4*, and *MdHY5* were cloned into the pGAD424 vector to generate MdICE1-pGAD, MdCBF1-pGAD, MdCBF2-pGAD, MdCBF3-pGAD, MdMYB23-pGAD, MdMYB308L-pGAD, MdbHLH3-pGAD, MdbHLH33-pGAD, MdBBX37-pGAD, MdABI4-pGAD, and MdHY5-pGAD. The recombinant plasmids were genetically transformed into "Y2H Gold" yeast cells by the polyethylene glycol (PEG) method. The transformed yeast cells were cultured on selective medium, and the growth of yeast was observed. The primers used for vector construction are listed in Supplemental Table 1.

Experimental procedure S2

Pull down assays

For constructing MdPUB23-pET32a, full-length *MdPUB23* was amplified and cloned into the pET32a vector. Full-length *MdICE1* was cloned into the pGEX4T-1 vector to generate MdICE1-pGEX4T-1. The MdPUB23-HIS and MdICE1-GST fusion proteins were obtained by the isopropyl-β-d-thiogalactoside (IPTG) induction of *Escherichia coli* "BL21" (TransGen Biotech, Beijing, China). A glutathione purification kit (Thermo Fisher Scientific) was used for pulldown assays as described in the instructions. The Western blotting was performed by using GST and HIS antibodies (Abmart, Shanghai, China).

Experimental procedure S3

Bimolecular fluorescence complementation (BiFC) assays

Full-length *MdPUB23* and *MdICE1* were amplified and cloned into pSPYNE-35S/pUC-SPYNE andpSPYCE-35S/pUC-SPYCE vectors to generate MdPUB23-YFP^{N} and MdICE1-YFP^{C}, respectively. The recombinant plasmids were transformed into onion epidermal cells by *Agrobacterium*-mediated genetic transformation. The fluorescence signals in the onion epidermal cells were observed using a fluorescence microscope (FV3000, Olympus, Japan).

Experimental procedure S4

Coimmunoprecipitation (Co-IP) assays

Full-length *MdICE1* and *MdPUB23* were amplified and cloned into pRI101-GFP and pCXSN-MYC vectors, respectively. The recombinant vectors were transformed into *Agrobacterium* and transiently transformed into apple calli by vacuuming. MdPUB23-MYC/GFP and MdPUB23-MYC/MdICE1-GFP transgenic apple calli were used for Co-IP assays. In brief, the ICE1-GFP and GFP proteins were immunoprecipitated from the above apple calli using an immunoprecipitation kit (Thermo Fisher Scientific). Western blot analysis was performed using MYC and GFP antibodies.
